# Haemodynamic flow abnormalities in bicuspid aortic valve disease improve with aortic valve replacement

**DOI:** 10.1186/1532-429X-17-S1-P330

**Published:** 2015-02-03

**Authors:** Malenka M Bissell, Margaret Loudon, Aaron T Hess, Victoria Stoll, Elizabeth Orchard, Stefan Neubauer, Saul G Myerson

**Affiliations:** 1 University of Oxford, Oxford, UK; 2 Cardiology, John Radcliffe Hospital, Oxford, UK

## Background

Bicuspid aortic valve disease (BAV) is associated with dilatation of the proximal aorta and abnormal flow patterns, particularly increased helical flow and changes in the aortic wall shear stress. These altered flow patterns may be partly responsible for the aortic dilation, though the aetiology is still unclear. Aortic valve replacement can modify the flow pattern in the proximal aorta (potentially to normal) and could thus have an effect on future aortic dilation. In this study, we aimed to assess the effect of different types of aortic valve replacement (AVR) on aortic flow patterns.

## Methods

We prospectively enrolled 69 participants: 23 BAV patients with prior AVR (10 mechanical, 6 bioprosthetic, 7 Ross procedure), 23 BAV patients with a native aortic valve and 23 healthy volunteers. All underwent 4D flow cardiovascular magnetic resonance.

## Results

The majority of patients with mechanical AVR or Ross showed a normalised flow pattern (70% and 57% respectively) with near normal rotational flow values (7.4±3.9 and 11.0±12.0mm^2^/s respectively; normal range -5 to +11 mm^2^/s); and reduced in-plane wall shear stress compared to native BAV (0.13±0.18N/m^2^ for mechanical AVR vs. 0.37±0.26N/m^2^ for native BAV, p<0.05). In contrast, all subjects with bioprosthetic AVR showed abnormal flow patterns (mainly marked right-handed helical flow), with similar rotational flow values to native BAV (25.3±15.0mm^2^/s and 20.1±11.0mm^2^/s respectively, p>0.05) and similar wall shear stress pattern. Data before and after AVR (n=13) supported these findings: mechanical AVR showed a significant reduction in rotational flow (29.3±15.1 to 7.9±4.2mm^2^/s, p<0.05) and in-plane wall shear stress (0.45±0.19 to 0.20±0.12N/m^2^, p<0.05), whereas these remained unchanged in the bioprosthetic AVR group.

## Conclusions

Abnormal flow patterns in BAV are significantly reduced after mechanical AVR or Ross procedure, but remain similar after bioprosthetic AVR. This is the first insight indicating that type of valve replacement may influence post-operative flow patterns, and could have important implications for future aortic growth.

## Funding

This study was funded by the British Heart Foundation.

**Figure 1:**
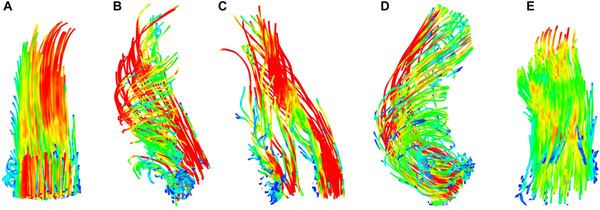
**Ascending aortic flow patterns;** A - healthy volunteer with a laminar flow pattern ; B - native bicuspid aortic valve disease with a right-handed helical flow pattern; C - AVR-mechanical with 2 laminar jets; D - AVR-tissue with a right-handed helical flow pattern; E - AVR-Ross with a laminar flow pattern

